# *Listeria monocytogenes*-Derived Membrane Vesicles Suppress Melanoma Growth via Macrophage Activation Involving NF-κB Signaling

**DOI:** 10.3390/microorganisms14051038

**Published:** 2026-05-03

**Authors:** Jiahui Liang, Mi Li, Shengxia Chen

**Affiliations:** Department of Laboratory Medicine, School of Medicine, Jiangsu University, Zhenjiang 212000, China; q893358108@163.com (J.L.); lmionly@163.com (M.L.)

**Keywords:** *Listeria monocytogenes*, membrane vesicles, macrophage activation, NF-κB signaling, melanoma, tumor immunotherapy

## Abstract

Melanoma is an aggressive skin cancer with rapid progression and high metastatic potential, and resistance to current therapies remains a major clinical challenge. In this study, *Listeria monocytogenes*-derived membrane vesicles (LM MVs) were isolated, characterized, and evaluated for their immunomodulatory and antitumor activities. LM MVs showed an average diameter of approximately 160 nm and contained multiple bacterial proteins, including listeriolysin O. In vitro, LM MVs promoted pro-inflammatory activation of RAW264.7 macrophages, as indicated by increased CD80/CD86 expression and enhanced transcription of inflammatory mediators. LM MV treatment was accompanied by IκB-α degradation and NF-κB p65 nuclear translocation, whereas pharmacological inhibition of NF-κB attenuated macrophage activation. In a macrophage–melanoma co-culture system, LM MVs-activated macrophages reduced the viability, migration, and invasion of B16 melanoma cells and increased tumor cell apoptosis. Additional inhibition and immunofluorescence analyses suggested that iNOS and TNF-α-associated mechanisms contributed to these tumor-suppressive effects. In a murine melanoma model, LM MVs significantly inhibited tumor growth without overt systemic toxicity, whereas macrophage depletion markedly weakened this effect. These findings indicate that LM MVs exert antitumor activity against melanoma, at least in part through macrophage activation involving NF-κB signaling.

## 1. Introduction

Melanoma is among the most aggressive forms of skin cancer and accounts for a disproportionate number of skin cancer-related deaths worldwide despite representing only a small fraction of all cutaneous malignancies [1,2,3]. Over the past decade, immune checkpoint inhibitors and targeted therapies have significantly improved clinical outcomes in patients with advanced melanoma [4]. However, a substantial proportion of patients fail to achieve durable benefit because of primary non-response or acquired therapeutic resistance [5,6,7,8]. The development of alternative or complementary strategies that can enhance antitumor immunity therefore remains an important priority.

The tumor microenvironment plays a central role in melanoma progression and treatment responsiveness. Among the immune cell populations present within tumors, macrophages are particularly abundant and display marked functional plasticity [9,10]. In response to distinct microenvironmental cues, macrophages may adopt phenotypic states broadly described as classically activated, pro-inflammatory macrophages and alternatively activated, immunoregulatory macrophages [11]. Pro-inflammatory macrophages generally exhibit tumor-suppressive properties through the production of inflammatory mediators and support of antitumor immunity, whereas immunoregulatory macrophages are often associated with tumor progression, angiogenesis and immune suppression [12]. Accordingly, therapeutic strategies aimed at promoting a more pro-inflammatory macrophage phenotype are increasingly being explored in cancer immunotherapy [13].

*Listeria monocytogenes* is a Gram-positive facultative intracellular bacterium well known for its ability to stimulate robust innate and adaptive immune responses [14]. Because it can infect professional antigen-presenting cells and promote antigen presentation through both major histocompatibility complex (MHC) class I and class II pathways, attenuated *Listeria* strains have been investigated as vectors for cancer immunotherapy [14,15]. In addition, *Listeria monocytogenes* itself has been reported to exert antitumor effects in certain contexts. Nevertheless, the use of live bacterial therapeutics remains constrained by biosafety concerns, including infection risk, residual virulence, and inter-individual variability in host responses.

Bacterial membrane vesicles are nanoscale extracellular vesicles naturally released during bacterial growth, typically ranging from 20 to 400 nm in diameter [16]. These vesicles contain diverse bacterial components, including proteins, lipids, and pathogen-associated molecular patterns, enabling them to interact with host cells and modulate immune responses [17]. Importantly, because membrane vesicles are non-replicative, they may avoid several of the safety concerns associated with live bacterial therapy [18,19]. Increasing evidence suggests that bacterial membrane vesicles may serve as effective immunomodulators and may have utility as vaccine platforms or antitumor agents [19,20,21].

Despite growing interest in bacterial membrane vesicles, the immunomodulatory and antitumor potential of *Listeria monocytogenes*-derived membrane vesicles has not been fully characterized. In particular, whether these vesicles can activate macrophages and thereby influence melanoma progression remains unclear. In the present study, we isolated and characterized membrane vesicles derived from *Listeria monocytogenes* and investigated their effects on macrophage activation and melanoma growth in vitro and in vivo.

## 2. Materials and Methods

### 2.1. Bacterial Strain and Culture Conditions

The *Listeria monocytogenes* EGD strain was kindly provided by Prof Yanna Shen (Tianjin Medical University, China). Bacteria were cultured in brain heart infusion (BHI) broth (Beijing Land Bridge Technology Co., Ltd., Beijing, China) at 37 °C with shaking until the optical density at 600 nm reached 1.0–1.2.

### 2.2. Isolation and Purification of Membrane Vesicles

Bacterial cultures were centrifuged at 6000× *g* for 20 min at 4 °C to remove bacterial cells. The resultant supernatant was concentrated using ultrafiltration in a tube with a 100 kDa retention capacity after being filtered using a 0.45 µm membrane (Millipore Corporation, Bedford, MA, USA).

The concentrated samples were ultracentrifuged at 150,000× *g* for 3 h at 4 °C (Beckman Coulter, Brea, CA, USA). The crude MV pellets were resuspended in sterile phosphate-busffered saline (PBS) and subjected to a second ultracentrifugation step under the same conditions for further purification. Final MV pellets were resuspended in sterile PBS, passed through a 0.22 μm filter, aliquoted, and stored at −80 °C until use. Protein concentrations were determined using a bicinchoninic acid (BCA) protein assay kit (Thermo Fisher Scientific, Waltham, MA, USA) according to the manufacturer’s instructions.

To verify the sterility of the LM MV preparation, an aliquot of the final vesicle suspension was inoculated into fresh BHI broth and incubated at 37 °C with shaking for 24 h. No bacterial growth was observed, indicating that the purified LM MVs were free of detectable viable bacteria.

### 2.3. Characterization of LM MVs

#### 2.3.1. Nanoparticle Tracking Analysis

The size distribution and concentration of LM MVs were analyzed using a ZetaView Twin PMX220 nanoparticle tracking analyzer (Particle Metrix GmbH, Inning am Ammersee, Germany).

#### 2.3.2. Transmission Electron Microscopy

For transmission electron microscopy (TEM; HT7800, Hitachi, Tokyo, Japan), LM MV samples were placed on copper grids and negatively stained with 2% uranyl acetate. After drying, the samples were examined using an HT7800 transmission electron microscope (Hitachi, Tokyo, Japan).

#### 2.3.3. SDS–PAGE Analysis of LM MVs Proteins

To evaluate the protein profile of LM MVs, total bacterial proteins were extracted from *Listeria monocytogenes* using a lysozyme-ultrasonication-TCA-acetone precipitation method [22]. Protein samples were separated on 10% precast gels (Vazyme, Biotech Co., Ltd., Nanjing, China). Prepared LM MV samples and bacterial protein samples were mixed with protein loading buffer and denatured by boiling prior to electrophoresis. Each sample (20 μL) was loaded onto the gel. Electrophoresis was performed at 60 V in the stacking gel, and when the samples reached the interface between the stacking and separating gels, the voltage was increased to 120 V until the run was completed. After electrophoresis, the gels were immersed in Coomassie Brilliant Blue staining solution and gently shaken for 1 h. The staining solution was then discarded, and the gels were destained with destaining solution, which was replaced every 30 min until the background became clear.

#### 2.3.4. Identification of LLO Protein

To identify listeriolysin O (LLO), LM MV protein samples were subjected to Western blot analysis. After SDS–PAGE electrophoresis, the separating gel was removed. The PVDF membrane was activated in methanol for 15 min, and proteins were then transferred onto the membrane under ice-bath conditions at a constant current of 260 mA for 2 h. After transfer, the membrane was blocked in blocking buffer (5% non-fat milk in TBST) on a shaker at room temperature for 2 h. The membrane was then washed with TBST for 10 min at room temperature, and this washing step was repeated three times.

The blocked membrane was incubated overnight at 4 °C with anti-LLO primary antibody (Abcam, Cambridge, UK, ab200538, 1:5000). On the following day, the membrane was washed with TBST for 10 min at room temperature, and this step was repeated three times. The membrane was then incubated with goat anti-rabbit IgG (H + L) secondary antibody (CWbio, Beijing, China, 1:5000) at room temperature for 2 h on a shaker, followed by three washes with TBST for 10 min each. Finally, the HRP chemiluminescent substrate (Treyo Biotech, Shanghai, China) was mixed at a 1:1 ratio, and the membrane was exposed for signal detection and imaging to determine the presence of the LLO band.

### 2.4. Cell Culture

RAW264.7 murine macrophages were obtained from the Cell Bank of the Institutes for Biological Sciences (Shanghai, China). B16 murine melanoma cells were purchased from iCell Bioscience (Shanghai) Co., Ltd., Shanghai, China. Cells were maintained in Dulbecco’s modified Eagle medium (DMEM) supplemented with 10% fetal bovine serum (FBS) at 37 °C in an incubator containing 5% CO_2_.

### 2.5. Transwell Co-Culture Experiments

To assess whether LM MV-activated macrophages exert antitumor effects on melanoma cells, a Transwell co-culture system was established. RAW264.7 macrophages were seeded into the lower chambers of 6-well Transwell plates (0.4 μm pore size, Corning Incorporated, Corning, NY, USA) at a density of 5 × 10^4^ cells per well, whereas B16 melanoma cells were seeded into the upper chambers at a density of 1 × 10^5^ cells per well. After overnight attachment, the medium in the lower chambers was replaced with fresh DMEM containing the indicated treatments, and the upper chambers were inserted to initiate co-culture. Cells were then cultured for 48 h at 37 °C in 5% CO_2_.

For dose-dependent co-culture experiments, RAW264.7 cells in the lower chambers were treated with PBS or LM MVs at concentrations of 10, 20, or 50 μg/mL. For inhibitor-related co-culture experiments, RAW264.7 cells in the lower chambers were divided into the following groups: PBS group, LM MV group (50 μg/mL), iNOS inhibitor + LM MVs group, TNF-α inhibitor + LM MVs group, and combined inhibitors + LM MVs group. The iNOS inhibitor 1400W dihydrochloride (Shandong Sparkjade Biotechnology Co., Ltd., Jinan, China) and the TNF-α inhibitor R-7050 (SparkJade, China) were both used at a final concentration of 10 μM and were added 1 h before LM MV treatment. LM MVs and inhibitors were added to the lower chambers containing RAW264.7 cells.

### 2.6. Cell Viability Assay

RAW264.7 macrophages were seeded into 96-well plates at a density of 5 × 10^3^ cells per well and cultured overnight. Cells were then treated with different concentrations of LM MVs for 48 h. Subsequently, 10 μL of CCK-8 reagent (Vazyme Biotech Co., Ltd., Nanjing, China) was added to each well, and cells were incubated for an additional 2 h at 37 °C. Absorbance was measured at 450 nm using a Cytation 5 microplate reader (BioTek Instruments, Winooski, VT, USA). PBS control wells and blank wells containing medium and CCK-8 reagent without cells were included, and the absorbance values were interpreted after background correction relative to the corresponding control groups. Three independent experiments were performed in triplicate.

For direct treatment experiments, B16 cells were seeded into 96-well plates at a density of 5 × 10^3^ cells per well and cultured overnight. The cells were then treated with the indicated concentrations of LM MVs for 48 h. Cell viability was subsequently measured using the same CCK-8 protocol. In parallel, apoptosis of B16 cells after direct LM MV treatment was assessed as described below.

For co-culture experiments, B16 cells were collected from the upper chambers after 48 h, washed with PBS, dissociated by trypsinization, and reseeded into 96-well plates at a density of 5 × 10^3^ cells per well. After 48 h of attachment, cell viability was measured using the same CCK-8 protocol.

### 2.7. Flow Cytometry Analysis

For macrophage polarization analysis, RAW264.7 cells were seeded into 6-well plates at a density of 1 × 10^5^ cells/well and allowed to attach for 24 h. The cells were then treated with 50 μg/mL LM MVs for 48 h, harvested, and stained with PE-conjugated anti-CD80 antibody and APC-conjugated anti-CD86 antibody (Elabscience Biotechnology Co., Ltd., Wuhan, China) for 30 min at 4 °C in the dark.

For apoptosis analysis in the co-culture system, two sets of experiments were performed. In the dose-dependent experiment, RAW264.7 cells in the lower chambers were treated with PBS or LM MVs at concentrations of 10, 20, or 50 μg/mL. In the inhibitor-related experiment, RAW264.7 cells in the lower chambers were divided into the following groups: Control group, LM MVs group (50 μg/mL), iNOS inhibitor + LM MVs group, TNF-α inhibitor + LM MVs group, and combined inhibitors + LM MVs group. The iNOS inhibitor 1400W dihydrochloride (SparkJade, China) and the TNF-α inhibitor R-7050 (SparkJade, China) were both used at a final concentration of 10 μM and were added 1 h before LM MV treatment. After 48 h of co-culture, B16 cells collected from the upper chambers were stained using an Annexin V-FITC/PI Apoptosis Detection Kit (Vazyme Biotech, China) according to the manufacturer’s instructions.

All samples were analyzed using a CytoFLEX flow cytometer (Beckman Coulter, Brea, CA, USA), and data were processed with FlowJo software (version 10.0.7r2; FlowJo LLC, Ashland, OR, USA). The flow cytometry results were interpreted in combination with other experimental findings.

### 2.8. Quantitative Real-Time PCR

Total RNA was extracted from RAW264.7 cells using TRIzol reagent (Vazyme, China) and reverse-transcribed into cDNA using HiScript Q RT SuperMix for qPCR (Vazyme, China). Quantitative real-time PCR (qRT-PCR) was performed using AceQ qPCR SYBR Green Master Mix (Vazyme, China) on a StepOnePlus Real-Time PCR System (Applied Biosystems, Foster City, CA, USA). Relative mRNA expression was calculated using the 2^−ΔΔCt^ method with *GAPDH* as the internal control. The primer sequences used for real-time PCR are listed in Table 1.

### 2.9. Immunofluorescence Staining

RAW264.7 cells were seeded on glass coverslips and treated with LM MVs for 1 h. Cells were fixed with 4% paraformaldehyde, permeabilized with 0.3% Triton X-100, and blocked with 5% bovine serum albumin (BSA). Cells were then incubated overnight at 4 °C with rabbit anti-p65 antibody (1:100, Cell Signaling Technology, Danvers, MA, USA), followed by Alexa Fluor 488-conjugated goat anti-rabbit IgG (1:500, Beyotime Biotechnology, Shanghai, China) for 1 h at room temperature. Nuclei were counterstained with DAPI. Images were captured using a Ti2-U fluorescence inverted microscope (Nikon, Tokyo, Japan). The proportion of cells showing predominant nuclear p65 staining was quantified.

### 2.10. NF-κB Inhibition Assay

RAW264.7 cells were pretreated with JSH-23 (20 μM) for 2 h and then stimulated with LM MVs (50 μg/mL). For analysis of p65 nuclear translocation, cells were collected after 1 h of LM MV stimulation. For flow cytometric assessment of macrophage polarization, cells were collected after 48 h of LM MV treatment.

### 2.11. Proteinase K Treatment

To evaluate whether proteinaceous components contribute to the macrophage-polarizing activity of LM MVs, LM MVs were subjected to proteinase K treatment before stimulation. For the active proteinase K treatment group, LM MVs (50 μg/mL) were incubated with proteinase K (100 μg/mL) at 37 °C for 2 h. For the heat-inactivated proteinase K control group, proteinase K was first heat-inactivated at 95 °C for 10 min and then incubated with LM MVs under the same conditions. Untreated LM MVs served as the control. The resulting preparations were subsequently used to stimulate RAW264.7 cells for polarization experiments.

### 2.12. Western Blot Analysis

Cells were lysed using RIPA lysis buffer supplemented with PMSF at a ratio of 99:1 (RIPA:PMSF = 99:1). Protein samples were collected, and protein concentrations were determined using a bicinchoninic acid (BCA) assay. For analysis of NF-κB signaling, cytoplasmic and nuclear proteins were extracted using a Nuclear and Cytoplasmic Protein Extraction Kit (Biosharp, Hefei, China) according to the manufacturer’s instructions.

Equal amounts of total, cytoplasmic, or nuclear proteins were mixed with protein loading buffer and denatured by boiling, followed by separation on 10% SDS-PAGE gels. Electrophoresis was performed at 60 V in the stacking gel, and when the samples entered the separating gel, the voltage was increased to 120 V until the run was completed. Proteins were then transferred onto PVDF membranes (Millipore, Burlington, MA, USA). The PVDF membranes were pre-activated with methanol for 15 s before use. Transfer was performed at a constant current of 260 mA for 2 h. After transfer, the membranes were blocked with 5% non-fat milk in TBST at room temperature for 2 h and then incubated overnight at 4 °C with primary antibodies diluted in 5% BSA. For total protein analysis, primary antibodies against cleaved caspase-3, BAX, and Bcl-2 were used. For NF-κB signaling analysis, primary antibodies against p65 and IκB-α were used. The antibodies used in this study are listed in Table 2. After washing, the membranes were incubated with HRP-conjugated goat anti-rabbit IgG secondary antibody (1:5000, CWBio, Beijing, China) for 2 h at room temperature. Protein bands were visualized using an enhanced chemiluminescence reagent (Treyo Biotech, Shanghai, China). Band intensities were quantified using ImageJ 1.54f software. GAPDH was used as the internal reference for total and cytoplasmic proteins, whereas Lamin B1 was used as the internal reference for nuclear proteins.

### 2.13. Wound Healing Assay

After 48 h of co-culture, B16 cells were collected from the upper chambers and seeded into 6-well plates at a density of 2 × 10^5^ cells per well. When cells reached approximately 90% confluence, a linear wound was created using a sterile 200 μL pipette tip. Detached cells were removed by washing with PBS. Cells were then cultured in serum-free medium, and wound closure was photographed at 0 h, 24 h, and 48 h using an inverted microscope. The wound area was quantified using ImageJ software.

### 2.14. Transwell Migration and Invasion Assays

After co-culture, B16 cells were collected and resuspended in serum-free DMEM. For migration assays, 2 × 10^4^ cells in 200 μL serum-free medium were seeded into the upper chambers of 24-well Transwell inserts (8 μm pore size, Corning, USA), while the lower chambers contained 600 μL DMEM supplemented with 10% FBS.

For invasion assays, the upper chambers were pre-coated with Matrigel (1:8 dilution in serum-free medium; BD Biosciences, Franklin Lakes, NJ, USA) and incubated at 37 °C for 2 h. Subsequently, 2 × 10^4^ cells were seeded into the upper chambers. After 48 h, cells remaining on the upper surface were removed with a cotton swab. Cells that had migrated or invaded to the lower surface were fixed with 4% paraformaldehyde and stained with 0.1% crystal violet. Stained cells were imaged and counted in five randomly selected fields per well.

### 2.15. Colony Formation Assay

A colony formation assay was performed to evaluate the effect of soluble factors released from LM MV-activated macrophages on the long-term proliferative capacity of B16 cells. RAW264.7 cells were seeded into 6-well plates at a density of 5 × 10^4^ cells/well and cultured for 24 h until attachment and stabilization. The cells were then divided into the following five groups: PBS group, LM MVs group (50 μg/mL), iNOS inhibitor + LM MVs group, TNF-α inhibitor + LM MVs group, and combined inhibitors + LM MVs group. The iNOS inhibitor 1400W dihydrochloride (SparkJade, China) and the TNF-α inhibitor R-7050 (SparkJade, China) were both used at a final concentration of 10 μM and were added 1 h before LM MV treatment. RAW264.7 cells were subsequently treated with 50 μg/mL LM MVs for 48 h.

After treatment, culture supernatants were collected and centrifuged to remove cellular debris. The resulting conditioned media were mixed with fresh complete medium at a ratio of 1:1 before use.

Meanwhile, B16 cells were seeded into 6-well plates at a density of 1 × 10^3^ cells/well. After 24 h of attachment, the corresponding conditioned media from each RAW264.7 treatment group were added to the B16 cells. The conditioned medium was refreshed every 3 days. After incubation for 7–10 days, the culture medium was discarded, and the cells were gently washed with PBS, fixed with 4% paraformaldehyde, and stained with 0.1% crystal violet. After washing and air drying, colony formation was photographed and counted. A cluster containing more than 50 cells was defined as a colony. Colony numbers were analyzed using ImageJ software or manual counting.

### 2.16. Animal Model

To establish the melanoma model, B16 cells (1 × 10^6^ cells in 100 μL PBS) were subcutaneously injected into the dorsal flank of male C57BL/6J mice (6 weeks old; *n* = 3 per group). Mice were randomly assigned to three groups: PBS control, LM MV treatment, and macrophage depletion plus LM MV treatment. LM MVs were administered intraperitoneally at a dose of 50 μg per mouse once daily for 10 consecutive days. Tumor volume was measured daily using calipers and calculated as follows:

Tumor volume = length × width^2^ × 0.5.

Body weight was also recorded daily throughout the treatment period.

At the end of the treatment period, mice were euthanized, and tumors and major organs, including the heart, liver, spleen, lungs, and kidneys, were collected. Tumor tissues were used for immunohistochemical and immunofluorescence analyses, while major organs were collected for histopathological evaluation.

To assess whether macrophages contributed to the antitumor effect of LM MVs, clodronate liposomes (LipoCLO, #40337ES08, Yeasen, Shanghai, China) were used for in vivo macrophage depletion. Mice in the macrophage depletion group received 100 μL clodronate liposomes (5 mg/mL) by intraperitoneal injection 1 day before tumor inoculation, followed by repeated administration every 4 days throughout the experiment.

All animal experiments were approved by the Experimental Animal Ethics Committee of the Experimental Animal Center of Jiangsu University (approval No. 15261) and conducted in accordance with institutional guidelines.

### 2.17. Histopathological Analysis of Major Organs

At the end of the animal experiment, major organs, including the heart, liver, spleen, lungs, and kidneys, were collected and fixed in 4% paraformaldehyde at room temperature for 24 h. After fixation, tissues were processed through a graded ethanol series for dehydration, embedded in paraffin, and sectioned at 4 μm thickness. The sections were then stained with hematoxylin and eosin (H&E) according to standard histological procedures. Histopathological changes in the major organs were examined under a light microscope to evaluate potential systemic toxicity of LM MV treatment.

### 2.18. Immunohistochemistry

Tumor tissues and major organs were fixed in 4% paraformaldehyde, embedded in paraffin, and sectioned at 5 μm thickness. Sections were deparaffinized, rehydrated, and subjected to antigen retrieval. After blocking with 5% goat serum, sections were incubated overnight at 4 °C with primary antibodies against CD86, Ki67, and cleaved Caspase-3. After washing, sections were incubated with HRP-conjugated secondary antibodies, developed with DAB substrate, and counterstained with hematoxylin. Images were captured using a light microscope (Olympus, Tokyo, Japan). Positive cells were quantified in five randomly selected fields per section.

### 2.19. Immunofluorescence Staining of Tumor Tissues

To assess the presence of macrophages expressing iNOS and TNF-α in tumor tissues, double immunofluorescence staining was performed. Tumor tissues were fixed in 4% paraformaldehyde, paraffin-embedded, and sectioned at 5 μm. After deparaffinization and rehydration, antigen retrieval was performed using citrate buffer. Sections were then blocked with 5% bovine serum albumin for 1 h at room temperature. The sections were incubated overnight at 4 °C with the following primary antibody combinations: anti-F4/80 plus anti-iNOS, or anti-F4/80 plus anti-TNF-α. After washing with PBS, sections were incubated with species-appropriate fluorophore-conjugated secondary antibodies for 1 h at room temperature in the dark. Nuclei were counterstained with DAPI. Fluorescence images were captured using a fluorescence microscope (Olympus).

### 2.20. Statistical Analysis

All data are presented as mean ± SEM from at least three independent experiments. Statistical analyses were performed using GraphPad Prism 8.0. Comparisons between two groups were conducted using Student’s *t*-test, whereas comparisons among multiple groups were analyzed by one-way or two-way ANOVA followed by Tukey’s post hoc test. A value of *p* < 0.05 was considered statistically significant.

## 3. Results

### 3.1. Isolation and Characterization of Listeria monocytogenes Membrane Vesicles

**Figure 1:**
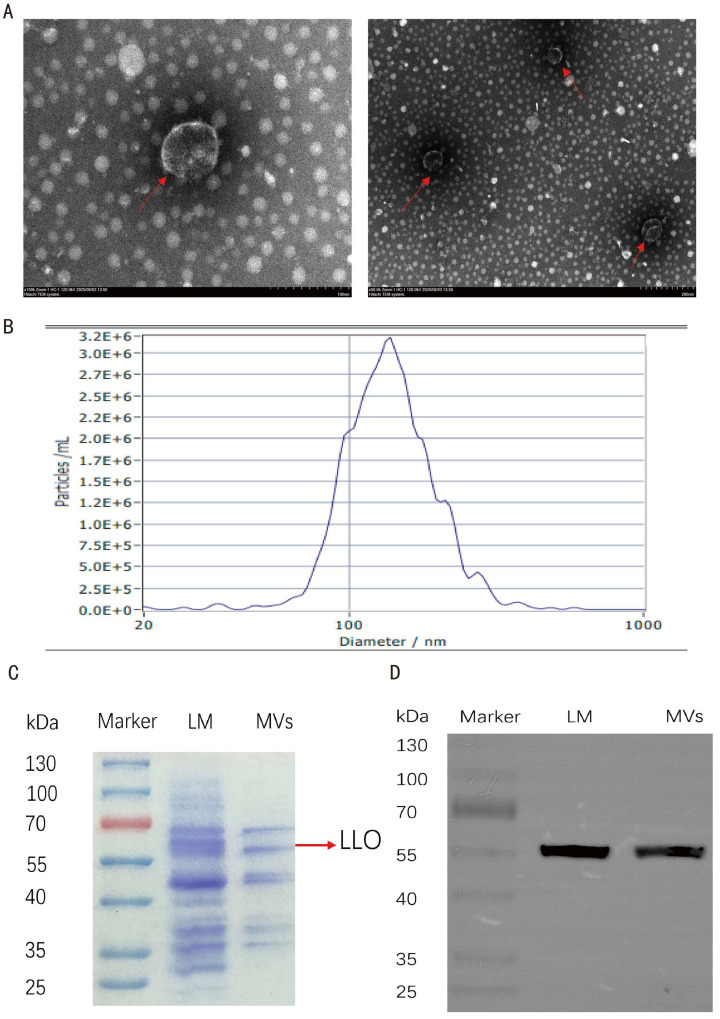
Isolation and characterization of *Listeria monocytogenes*-derived membrane vesicles (LM MVs). (**A**) Transmission electron microscopy image of purified LM MVs. Red arrows indicate representative LM MVs; (**B**) Nanoparticle tracking analysis showing the size distribution of LM MVs; (**C**) SDS–PAGE analysis of protein profiles in *Listeria monocytogenes* whole-cell lysates and LM MVs. The red arrow indicates the putative LLO band at approximately 55 kDa; (**D**) Western blot analysis showing the presence of LLO in LM MVs. Scale bars = 100 nm (left) and 200 nm (right).

The isolated LM MVs were first characterized by transmission electron microscopy, which revealed spherical membranous structures consistent with vesicles (Figure 1A). Nanoparticle tracking analysis showed that the average diameter of LM MVs was approximately 164 nm (Figure 1B). The protein concentration of the purified vesicle preparation was 2 mg/mL as determined by BCA assay. SDS–PAGE analysis revealed a prominent protein band between 55 and 70 kDa (Figure 1C). Western blot analysis further detected listeriolysin O (LLO) in the LM MV preparation, with an immunoreactive band at approximately 55 kDa (Figure 1D). The original images corresponding to Figure 1C,D are provided in Figure S1. Together, these results indicate successful isolation of LM MVs and confirm the presence of vesicle-associated bacterial proteins, including LLO.

### 3.2. LM MVs Promote Pro-Inflammatory Activation of Macrophages

**Figure 2:**
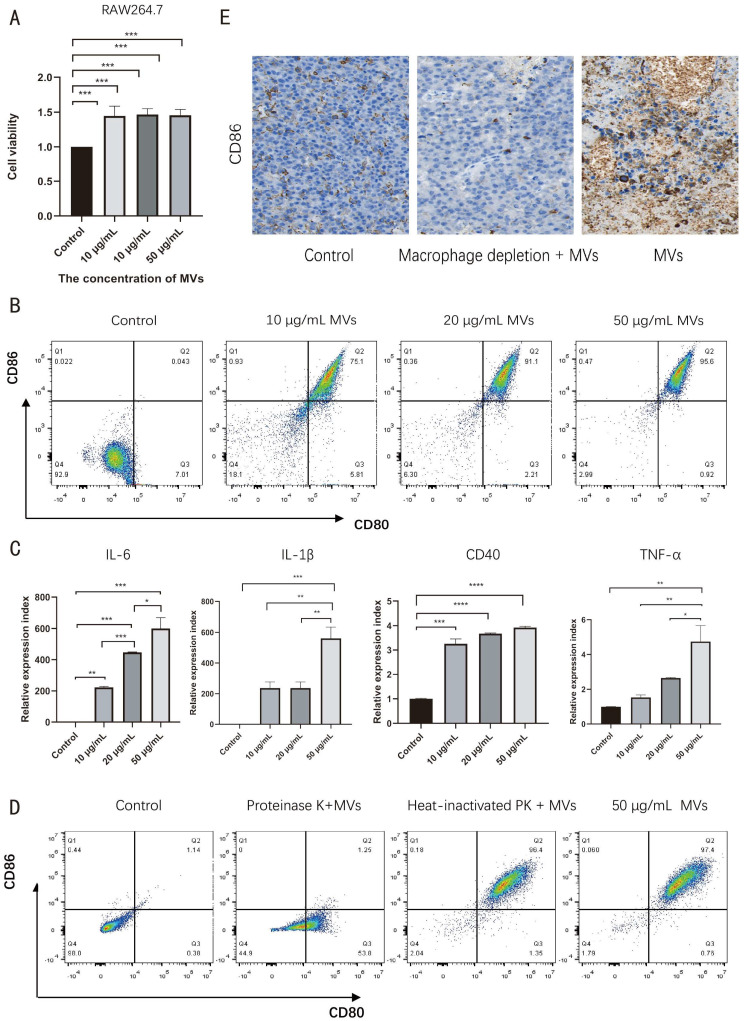
LM MVs promote pro-inflammatory activation of RAW264.7 macrophages. (**A**) CCK-8 assay of RAW264.7 cell viability after treatment with different concentrations of LM MVs for 48 h; (**B**) Flow cytometric analysis of CD80 and CD86 expression in RAW264.7 macrophages after LM MV treatment; (**C**) qRT-PCR analysis of *IL-6*, *IL-1β*, *CD40*, and *TNF-α* mRNA expression in RAW264.7 cells after LM MV stimulation; (**D**) Flow cytometric analysis of macrophage activation after stimulation with untreated LM MVs, proteinase K-treated LM MVs, or heat-inactivated proteinase K-treated LM MVs; (**E**) Immunohistochemical staining of CD86 in tumor tissues from different treatment groups. Data are presented as mean ± SEM from at least three independent experiments. One-way ANOVA with Tukey’s post hoc test. * *p* < 0.05, ** *p* < 0.01, *** *p* < 0.001, and **** *p* < 0.0001.

To investigate the effects of LM MVs on macrophages, RAW264.7 cells were treated with the indicated concentrations of LM MVs. CCK-8 assay showed no overt cytotoxicity under the tested conditions; in addition, LM MVs at 10, 20, and 50 μg/mL increased the CCK-8 signal relative to the control group (Figure 2A), suggesting enhanced cellular metabolic activity after stimulation. Flow cytometric analysis of CD80 and CD86 demonstrated that LM MVs promoted a CD80^+^/CD86^+^ macrophage phenotype at all tested concentrations, with the most pronounced effect observed at 50 μg/mL (Figure 2B). Consistently, qRT-PCR analysis showed that LM MV treatment significantly increased the mRNA levels of inflammatory mediators associated with macrophage activation, including *IL-6*, *IL-1β*, *CD40*, and *TNF-α* (Figure 2C).

To determine whether protein components of LM MVs contributed to this activity, LM MVs were pretreated with proteinase K before incubation with RAW264.7 cells. Flow cytometric analysis showed that proteinase K treatment markedly reduced LM MV-induced macrophage activation (Figure 2D), suggesting that proteinaceous vesicle components contribute to this immunostimulatory activity.

In addition, immunohistochemical analysis of tumor tissues showed increased CD86 staining after LM MV treatment (Figure 2E), and the corresponding original images are provided in Figure S3, consistent with enhanced pro-inflammatory immune features in vivo. Collectively, these findings suggest that LM MVs promote a pro-inflammatory macrophage-associated phenotype in vitro and are associated with enhanced pro-inflammatory immune features in vivo.

LM MVs promoted a pro-inflammatory macrophage-associated phenotype without overt cytotoxicity under the tested conditions. 

### 3.3. LM MVs Induce Macrophage Activation in Association with NF-κB Signaling

**Figure 3:**
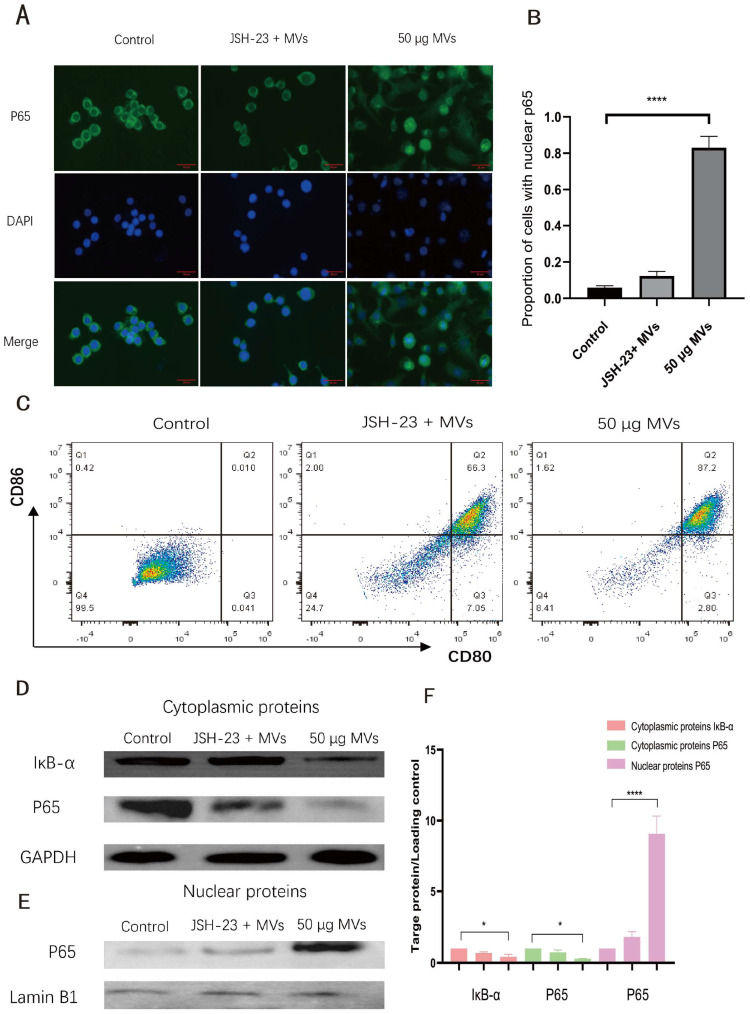
NF-κB signaling is involved in LM MV-induced macrophage activation. (**A**) Immunofluorescence staining of NF-κB p65 in RAW264.7 cells after treatment with PBS, LM MVs, or JSH-23 plus LM MVs. Nuclei were counterstained with DAPI; (**B**) Quantification of cells showing predominant nuclear p65 localization; (**C**) Flow cytometric analysis of CD80/CD86 double-positive RAW264.7 macrophages after LM MV stimulation in the presence or absence of JSH-23; (**D**) Western blot analysis of cytoplasmic IκB-α and nuclear p65 after LM MV stimulation for the indicated times; (**E**) Densitometric analysis of IκB-α and nuclear p65 protein levels. One-way ANOVA with Tukey’s post hoc test. * *p* < 0.05 and **** *p* < 0.0001. Scale bar = 25 µm.

To explore the mechanism underlying LM MV-induced macrophage activation, the subcellular localization of NF-κB p65 was examined by immunofluorescence staining. In control cells, p65 was predominantly localized in the cytoplasm. In contrast, LM MV treatment induced marked nuclear accumulation of p65, as evidenced by increased nuclear fluorescence intensity and a higher proportion of cells showing predominant nuclear p65 staining (Figure 3A). The original immunofluorescence images corresponding to Figure 3A are provided in Figure S4. Quantitative analysis showed that approximately 80% of macrophages exhibited nuclear p65 localization after LM MV stimulation (Figure 3B).

To assess whether NF-κB signaling contributed to LM MV-induced macrophage activation, RAW264.7 cells were pretreated with JSH-23 before LM MV stimulation. JSH-23 markedly reduced LM MV-induced nuclear accumulation of p65 (Figure 3A) and significantly decreased the proportion of CD80/CD86 double-positive macrophages after stimulation (Figure 3C). These data support the involvement of NF-κB signaling in LM MV-induced macrophage activation.

**Figure 4:**
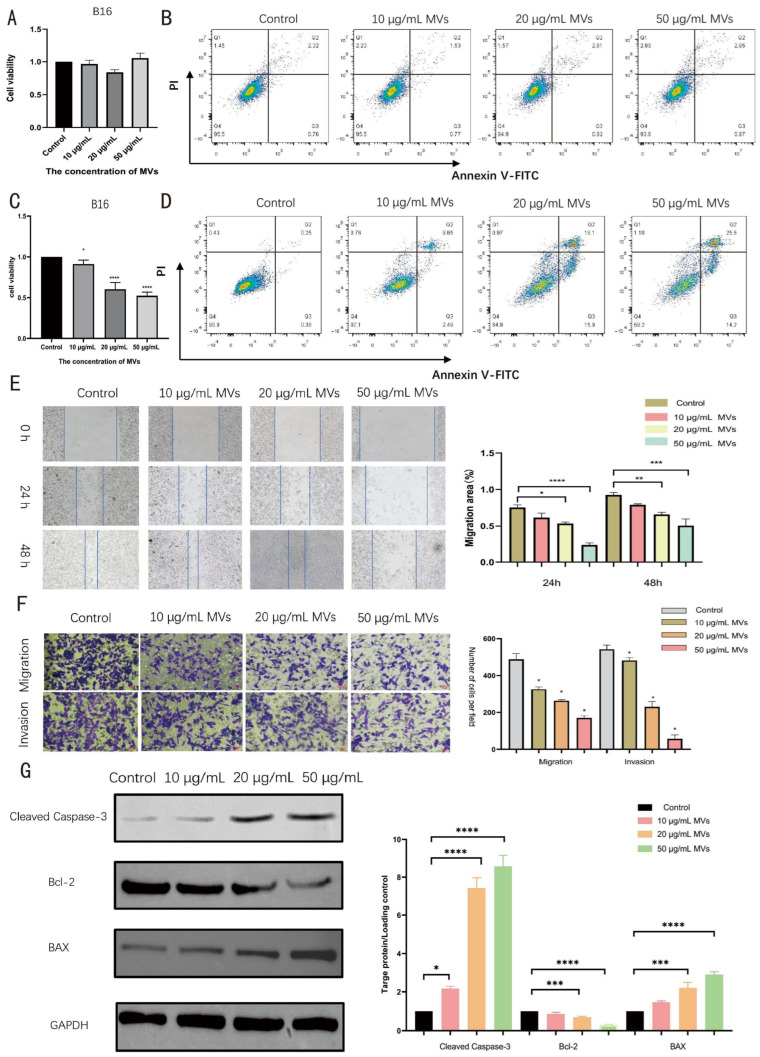
LM MV-activated macrophages suppress melanoma cell growth and malignant behavior in vitro. (**A**) CCK-8 assay of B16 cell viability after direct LM MV treatment for 48 h; (**B**) Flow cytometric analysis of apoptosis in B16 cells after direct LM MV treatment; (**C**) CCK-8 assay of B16 cell viability after co-culture with LM MV-activated macrophages; (**D**) Flow cytometric analysis of apoptosis in B16 cells after co-culture with LM MV-activated macrophages; (**E**) Wound healing assay showing the migratory capacity of B16 cells after co-culture; (**F**) Transwell migration and invasion assays of B16 cells after co-culture; (**G**) Western blot analysis of cleaved Caspase-3, BAX and Bcl-2 in B16 cells after co-culture with LM MV-activated macrophages. Data are presented as mean ± SEM from at least three independent experiments. One-way ANOVA with Tukey’s post hoc test. * *p* < 0.05, ** *p* < 0.01, *** *p* < 0.001, and **** *p* < 0.0001.

To further examine activation of this pathway, nuclear and cytoplasmic protein fractions were analyzed by Western blot. Following LM MV stimulation, cytoplasmic IκB-α levels decreased over time, whereas nuclear p65 levels increased progressively (Figure 3D,E). The original Western blot images corresponding to Figure 3E and Figure 4G are provided in Figure S2. Together, these findings support activation of canonical NF-κB signaling in macrophages following LM MV stimulation and suggest that this pathway contributes to the observed pro-inflammatory response.

LM MV stimulation induced IκB-α degradation and p65 nuclear translocation, whereas JSH-23 attenuated macrophage activation. Data are presented as mean ± SEM from at least three independent experiments. 

### 3.4. LM MVs Suppress Melanoma Cell Growth Indirectly Through Macrophage Activation

To determine whether LM MVs directly affected melanoma cells, B16 cells were treated with the indicated concentrations of LM MVs for 48 h. CCK-8 assay showed that direct LM MV treatment had no significant effect on B16 cell viability (Figure 4A). Likewise, flow cytometric analysis indicated that LM MVs alone did not cause a marked increase in apoptosis in B16 cells under the tested conditions (Figure 4B). These findings suggest that LM MVs do not exert substantial direct cytotoxic effects on B16 melanoma cells in this experimental setting.

A macrophage–melanoma co-culture system was then used to examine whether LM MVs could suppress tumor cell behavior indirectly through macrophage activation. In this system, LM MV treatment significantly reduced B16 cell viability, with the strongest inhibitory effect observed at 50 μg/mL (Figure 4C). Consistently, flow cytometric analysis showed that the apoptotic rate of B16 cells was markedly increased in the co-culture group treated with 50 μg/mL LM MVs (Figure 4D).

Wound healing assays further demonstrated that co-culture with LM MV-stimulated macrophages significantly impaired the migratory capacity of B16 cells (Figure 4E). Transwell assays confirmed that both migration and invasion of B16 cells were significantly reduced after co-culture with LM MV-activated macrophages (Figure 4F). The original images corresponding to the wound healing and Transwell assays are provided in Figures S5 and S6. In addition, Western blot analysis showed increased expression of cleaved Caspase-3 and BAX, together with decreased expression of Bcl-2, in B16 cells following co-culture with LM MV-stimulated macrophages (Figure 4G). The original Western blot images corresponding to Figure 3E and Figure 4G are provided in Figure S2. Taken together, these data indicate that LM MVs suppress melanoma cell growth and malignant behavior largely through indirect, macrophage-mediated effects.

Direct LM MV treatment had little effect on B16 cells, whereas LM MV-activated macrophages reduced B16 cell viability, increased apoptosis, and suppressed migration and invasion.

### 3.5. LM MVs Inhibit Melanoma Growth In Vivo with a Macrophage-Dependent Component

**Figure 5:**
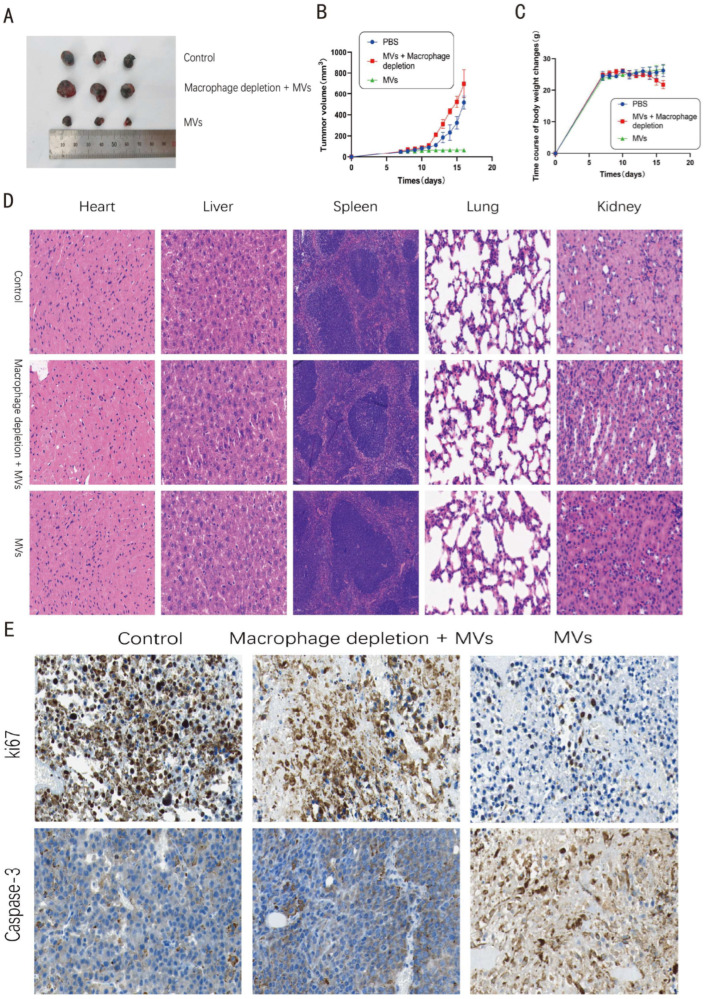
LM MVs inhibit melanoma growth in vivo in a macrophage-dependent manner. (**A**) Representative images of tumors excised from mice in the PBS, LM MVs, and macrophage depletion plus LM MVs groups; (**B**) Tumor growth curves during treatment; (**C**) Body weight changes during treatment; (**D**) Hematoxylin and eosin staining of major organs from different treatment groups; (**E**) Immunohistochemical staining of cleaved Caspase-3 and Ki67 in tumor tissues, with quantitative analysis.

To evaluate the antitumor activity of LM MVs in vivo, a B16 melanoma subcutaneous transplantation model was established. Mice were assigned to PBS control, LM MV treatment, and macrophage depletion plus LM MV treatment groups. At the end of the experiment, tumors excised from LM MV-treated mice were visibly smaller than those from PBS-treated controls (Figure 5A). Tumor growth curves showed that LM MV treatment significantly inhibited tumor growth from day 6 onward, and this effect was maintained throughout the observation period (Figure 5B). Notably, macrophage depletion markedly weakened the antitumor effect of LM MVs, supporting an important contribution of macrophages to this response.

To assess biosafety, body weight was monitored throughout the experiment. No significant differences in body weight were observed between LM MV-treated and control mice (Figure 5C). Moreover, hematoxylin and eosin staining of major organs, including the heart, liver, spleen, lungs, and kidneys, revealed no obvious pathological abnormalities in LM MV-treated mice under the tested conditions, suggesting the absence of overt systemic toxicity (Figure 5D).

Immunohistochemical analysis of tumor tissues showed that LM MV treatment significantly increased cleaved Caspase-3 staining and decreased Ki67 staining (Figure 5E), The original histological and immunohistochemical images corresponding to Figure 5D,E are provided in Figures S7 and S8. indicating enhanced tumor cell apoptosis and reduced proliferation in vivo. These results are consistent with the in vitro co-culture findings and further support the antitumor activity of LM MVs in this melanoma model.

### 3.6. iNOS and TNF-α Are Involved in the Antitumor Effects Mediated by LM MV-Activated Macrophages

**Figure 6:**
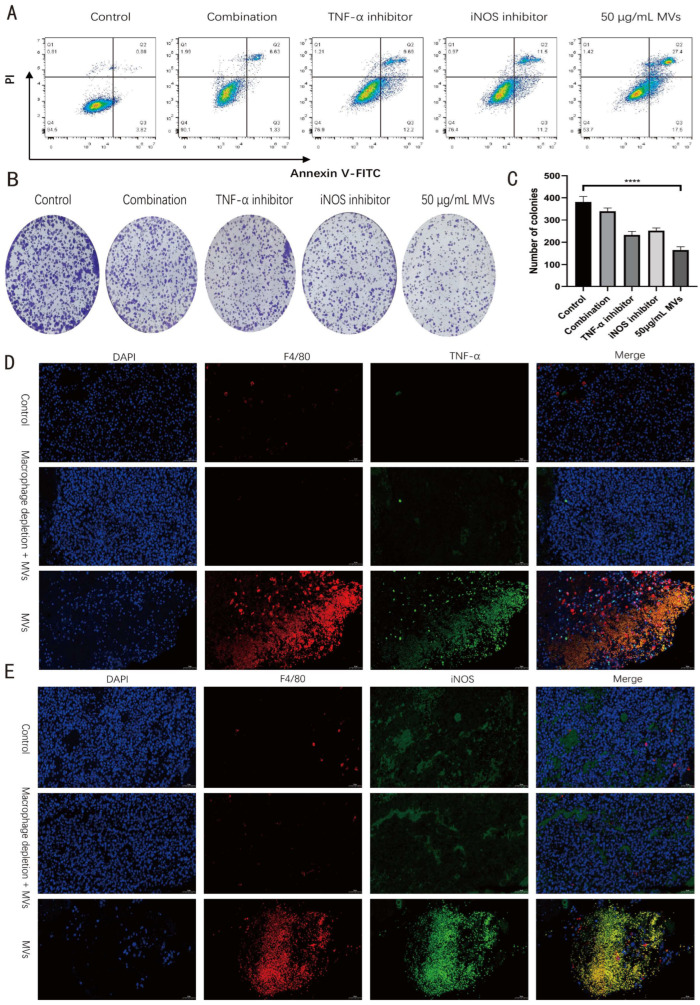
iNOS and TNF-α contribute to the antitumor effects mediated by LM MV-activated macrophages. (**A**) Flow cytometric analysis of apoptosis in B16 cells in the macrophage–melanoma co-culture system after treatment with LM MVs in the presence or absence of iNOS and/or TNF-α inhibitors; (**B**) Representative images of colony formation of B16 cells cultured with conditioned medium from differently treated RAW264.7 macrophages; (**C**) Quantification of colony numbers in the colony formation assay; (**D**) Representative double immunofluorescence staining images of F4/80 and iNOS in tumor tissues from different treatment groups; (**E**) Representative double immunofluorescence staining images of F4/80 and TNF-α in tumor tissues from different treatment groups. Data are presented as mean ± SEM from at least three independent experiments. One-way ANOVA with Tukey’s post hoc test. **** *p* < 0.0001. Scale bar = 50 µm.

To further investigate the downstream mediators responsible for the antitumor effects of LM MV-activated macrophages, inhibitor-based co-culture experiments were performed. Flow cytometric analysis showed that LM MVs markedly increased apoptosis of B16 cells in the macrophage–melanoma co-culture system compared with the control group. In contrast, inhibition of iNOS or TNF-α partially attenuated LM MV-induced B16 cell apoptosis, whereas combined inhibition produced a more pronounced reversal effect (Figure 6A). These findings suggest that iNOS- and TNF-α-associated pathways are involved in the pro-apoptotic effect exerted by LM MV-activated macrophages on melanoma cells.

Consistent with the apoptosis data, colony formation assays further demonstrated that conditioned medium derived from LM MV-stimulated RAW264.7 cells significantly suppressed the clonogenic growth of B16 cells. This inhibitory effect was partially restored when iNOS or TNF-α was inhibited, and combined inhibition led to a stronger recovery of colony-forming capacity (Figure 6B,C). These results indicate that iNOS and TNF-α contribute to the long-term growth-suppressive effect mediated by LM MV-activated macrophages.

To further assess whether these macrophage-associated effector molecules were increased in vivo, double immunofluorescence staining for F4/80 with iNOS or TNF-α was performed on tumor sections. Compared with the PBS group, LM MV treatment was associated with an increased presence of F4/80^+^iNOS^+^ and F4/80^+^TNF-α^+^ cells in tumor tissues (Figure 6D,E), and the corresponding original images are provided in Figures S9 and S10. suggesting enhanced accumulation or activation of macrophages expressing iNOS and TNF-α in the tumor microenvironment. These in vivo findings are consistent with the in vitro inhibitor experiments and further support the involvement of macrophage-associated iNOS and TNF-α in the antitumor activity of LM MVs.

Taken together, these results demonstrate that the antitumor effects of LM MV-activated macrophages are mediated, at least in part, through iNOS- and TNF-α-associated mechanisms, which contribute to enhanced tumor cell apoptosis and reduced clonogenic potential.

Inhibition of iNOS or TNF-α partially reversed the antitumor effects of LM MV-activated macrophages, whereas combined inhibition produced a stronger effect. In vivo immunofluorescence further showed an increased presence of F4/80^+^iNOS^+^ and F4/80^+^TNF-α^+^ cells after LM MV treatment.

## 4. Discussion

In the present study, we show that membrane vesicles derived from *Listeria monocytogenes* suppress melanoma growth in association with macrophage activation involving NF-κB signaling. Using a combination of in vitro macrophage assays, macrophage–tumor co-culture experiments, and an in vivo melanoma model, we found that LM MVs promoted a pro-inflammatory macrophage-associated phenotype and were associated with significant antitumor effects. Notably, LM MVs did not exert substantial direct cytotoxicity against B16 melanoma cells under the tested conditions, suggesting that their antitumor activity is mediated primarily through modulation of host immune cells rather than direct tumor cell killing.

Macrophages are highly plastic immune cells that play important roles in shaping the tumor microenvironment [23,24]. In many tumors, tumor-associated macrophages acquire phenotypes that support tumor growth, angiogenesis, and immune suppression. By contrast, pro-inflammatory macrophages can contribute to tumor control through the production of inflammatory mediators and by supporting broader antitumor immune responses [9,11,12]. In the present study, LM MVs increased the expression of CD80 and CD86 and enhanced the transcription of inflammatory mediators such as *IL-6*, *IL-1β*, and *TNF-α* in RAW264.7 macrophages, consistent with induction of a pro-inflammatory activation state. These findings are in line with previous studies showing that bacterial components and bacterial vesicles can activate innate immune signaling pathways and promote inflammatory macrophage responses [20,25].

Bacterial membrane vesicles have attracted increasing interest as immunomodulatory agents because they retain bioactive bacterial components while lacking replicative capacity. Vesicles derived from both Gram-negative and Gram-positive bacteria have been reported to activate innate immunity and, in some settings, enhance antitumor responses [16,17]. For example, Kim et al. showed that bacterial outer membrane vesicles can induce interferon-γ-dependent antitumor immunity in murine models [19]. Our results extend this concept by suggesting that vesicles derived from *Listeria monocytogenes* can stimulate macrophage activation and indirectly suppress melanoma progression.

Mechanistically, our data indicate that NF-κB signaling is involved in the macrophage response to LM MVs. LM MV stimulation was accompanied by degradation of IκB-α and nuclear translocation of p65, and pharmacological inhibition of NF-κB attenuated the induction of CD80/CD86-positive macrophages. These findings are consistent with the established role of NF-κB as a central regulator of inflammatory macrophage activation [26,27]. At the same time, our results should be interpreted with appropriate caution. Although they support a role for NF-κB in LM MV-induced macrophage activation, they do not exclude contributions from additional upstream receptors or parallel signaling pathways. Further studies will be needed to identify the pattern-recognition receptors and vesicle-associated components responsible for triggering this response.

An important observation in this study is that the antitumor effect of LM MVs appears to be largely indirect. Direct exposure of B16 cells to LM MVs did not significantly affect cell viability or apoptosis, whereas pronounced tumor-suppressive effects were observed when B16 cells were co-cultured with LM MV-stimulated macrophages. Moreover, macrophage depletion in vivo markedly weakened the therapeutic effect of LM MVs. Together, these data support a model in which macrophages are important mediators of LM MV-induced tumor suppression in this melanoma setting. Nevertheless, because the downstream effector molecules released by activated macrophages were not dissected here, the precise mechanisms by which macrophages restrain tumor cell growth, migration, and invasion remain to be clarified.

Building on this observation, the present study further suggests that the macrophage-mediated antitumor effect of LM MVs involves iNOS and TNF-α-associated mechanisms. In the inhibitor-based co-culture experiments, pharmacological inhibition of iNOS or TNF-α partially attenuated the ability of LM MV-stimulated macrophages to induce apoptosis in B16 cells, whereas combined inhibition produced a more pronounced reversal effect. Similarly, conditioned medium derived from LM MV-stimulated RAW264.7 cells markedly suppressed the clonogenic growth of B16 cells, and this inhibitory effect was partially relieved by inhibition of iNOS or TNF-α. These observations suggest that soluble mediators associated with inflammatory macrophage activation contribute to both acute tumor cell apoptosis and long-term suppression of proliferative potential.

Among the effector molecules produced by activated macrophages, iNOS and TNF-α are well-established mediators of inflammatory and antitumor responses [28]. iNOS catalyzes the production of nitric oxide, which can exert cytotoxic and cytostatic effects on tumor cells, whereas TNF-α functions as a pleiotropic inflammatory cytokine capable of promoting tumor cell death and reshaping the tumor microenvironment. In the present study, the partial reversal of LM MV-induced antitumor effects by iNOS or TNF-α inhibition suggests that these pathways contribute substantially to the functional output of LM MV-activated macrophages.

Our in vivo immunofluorescence data further support this interpretation. Compared with the PBS group, LM MV treatment was associated with an increased presence of F4/80^+^iNOS^+^ cells and F4/80^+^TNF-α^+^ cells in tumor tissues, suggesting enhanced accumulation or activation of macrophages expressing these effector molecules in the tumor microenvironment. These findings are consistent with the in vitro inhibitor experiments and strengthen the conclusion that LM MVs promote a pro-inflammatory, tumor-suppressive macrophage program. Nevertheless, these results should still be interpreted with caution. Because the inhibitor experiments were pharmacological in nature and the in vivo data were based on immunofluorescence staining, the present study does not establish iNOS and TNF-α as the sole downstream mediators of LM MV-induced antitumor activity. Future studies incorporating more direct genetic or molecular approaches will be required to define the relative contribution of these pathways more precisely.

We also detected listeriolysin O in the LM MV preparation. LLO is a cholesterol-dependent pore-forming toxin that plays a central role in *Listeria monocytogenes* virulence and intracellular survival [29]. Previous studies have shown that LLO can activate immune signaling pathways and induce cytokine production in macrophages [29,30]. In our study, proteinase K treatment reduced the macrophage-activating effect of LM MVs, suggesting that vesicle-associated proteins contribute to this activity. However, the present data do not establish LLO as the sole or dominant functional component. The inclusion of a heat-inactivated proteinase K control further supports the interpretation that the observed reduction was associated with proteolytic activity rather than the treatment procedure itself. Additional experiments using LLO-deficient vesicles, neutralizing strategies, or more detailed cargo analyses will be required to define the relative contributions of individual vesicle-associated molecules.

From a translational perspective, vesicle-based immunotherapeutic approaches may offer advantages over live bacterial therapy [31,32]. Although attenuated *Listeria* strains have been explored as cancer vaccine vectors, concerns regarding safety and infection risk have limited their broader application. In contrast, membrane vesicles retain immunostimulatory components while lacking the ability to replicate, which may improve safety. In the current study, LM MV treatment inhibited tumor growth without overt body weight loss or obvious histopathological damage in major organs, supporting the feasibility of this approach under the tested conditions. Even so, more comprehensive toxicological, pharmacokinetic, and biodistribution analyses will be necessary before clinical translation can be considered.

Several limitations of this study should be acknowledged. First, macrophage activation was primarily evaluated using the RAW264.7 cell line [33], and validation in primary macrophages will be necessary to confirm the generalizability of these findings. Second, although we observed increased CD86 staining in tumor tissues and attenuation of efficacy after clodronate-mediated macrophage depletion, in vivo immune profiling remained limited. More detailed analysis of macrophage populations and other immune cell subsets would help clarify how LM MVs reshape the tumor microenvironment. In addition, the efficiency of macrophage depletion was not directly quantified in the present study. Detailed documentation of the compensation settings used for the flow cytometry analysis was also not available, which may limit the technical precision of the FACS data interpretation. Third, key mechanistic inferences, including the involvement of NF-κB, iNOS, and TNF-α, were based primarily on pharmacological inhibition, and more definitive mechanistic approaches will be needed to confirm pathway dependence. Fourth, the upstream innate immune receptors that sense LM MVs were not investigated, and the specific vesicle-associated components responsible for the observed activity remain to be identified. Although LLO is a plausible contributor, other protein and non-protein cargoes may also participate.

Future studies should therefore focus on identifying the vesicle-associated components and upstream receptors responsible for macrophage activation, validating these findings in primary macrophages and additional melanoma models, and characterizing the in vivo immune response in greater detail. It will also be important to determine whether LM MVs can be combined with existing immunotherapies, particularly immune checkpoint blockade, and whether they can induce durable antitumor immune memory. Overall, our results indicate that LM MVs can activate macrophages and suppress melanoma growth in association with NF-κB signaling and downstream iNOS and TNF-α related mechanisms. These findings support further investigation of *Listeria monocytogenes*–derived membrane vesicles as a promising bacterial-derived immunomodulatory platform for melanoma therapy. 

## 5. Conclusions

In summary, our study demonstrates that membrane vesicles derived from *Listeria monocytogenes* suppress melanoma growth and promote pro-inflammatory macrophage activation in vitro and in vivo. These effects are associated with activation of NF-κB signaling in macrophages and are markedly weakened by macrophage depletion in the melanoma model examined here. The antitumor activity of LM MVs also appears to involve iNOS and TNF-α-associated macrophage effector mechanisms. Under the experimental conditions used, LM MVs inhibited tumor growth without overt systemic toxicity. Collectively, these findings support LM MVs as a potential bacterial-derived immunomodulatory candidate for melanoma therapy and provide a basis for future studies aimed at optimizing vesicle-based antitumor strategies.

## Figures and Tables

**Table 1:** Primers used for real-time PCR.

Gene	Forward (5′–3′)	Reverse (5′–3′)
*IL-6*	TAGTCCTTCCTACCCCAATTTCC	TTGGTCCTTAGCCACTCCTTC
*IL-1β*	GCAACTGTTCCTGAACTCAACT	ATCTTTTGGGGTCCGTCAACT
*CD40*	TGTCATCTGTGAAAAGGTGGTC	ACTGGAGCAGCGGTGTTATG
*TNF-α*	GGCCTCCCTCTCATCAGTTC	GGTGGTTTGCTACGACGTG
*GAPDH*	GGTTGTCTCCTGCGACTTCA	TGGTCCAGGGTTTCTTACTCC

**Table 2:** Antibodies used in this study.

Antibodies	Catalog Number	Host	Clonality	Working Dilution	Supplier
Cleaved Caspase-3	ET1602-47	Rabbit	Mono	1:1000	HuaBio (Woburn, MA, USA)
Bcl-2	ET1603-11	Rabbit	Mono	1:1000	HuaBio
BAX	ET1603-34	Rabbit	Mono	1:1000	HuaBio
GAPDH	CW0100M	Rabbit	Mono	1:50,000	CWbio
LLO	ab200538	Rabbit	Mono	1:5000	abcam
IκB-α	ET1603-6	Rabbit	Mono	1:5000	HuaBio
Lamin B1	ET1606-27	Rabbit	Mono	1:50,000	HuaBio
p65	8242T	Rabbit	Mono	1:1000	Cell Signaling
Goat Anti-Rabbit IgG HRP Conjugated	CW0103S	Goat	poly	1:5000	CWbio

## Data Availability

The original contributions presented in this study are included in this article. Further inquiries can be directed to the corresponding author.

## Supplementary Materials

The following supporting information can be downloaded at: https://www.mdpi.com/article/10.3390/microorganisms14051038/s1, Figure S1: Original images corresponding to Figure 1C,D; Figure S2: Original Western blot images corresponding to Figure 3E and Figure 4G; Figure S3: Original CD86 immunohistochemistry images corresponding to Figure 2E; Figure S4: Original p65 immunofluorescence images corresponding to Figure 3A; Figure S5: Original wound healing assay images corresponding to Figure 4E; Figure S6: Original Transwell migration and invasion assay images corresponding to Figure 4F; Figure S7: Original hematoxylin and eosin staining images of major organs corresponding to Figure 5D; Figure S8: Original immunohistochemistry images of cleaved Caspase-3 and Ki67 corresponding to Figure 5E; Figure S9: Original F4/80 and TNF-α immunofluorescence images corresponding to Figure 6D; Figure S10: Original F4/80 and iNOS immunofluorescence images corresponding to Figure 6E.

## Abbreviations

The following abbreviations are used in this manuscript:

LM MVs	*Listeria monocytogenes*-derived membrane vesicles
NF-κB	Nuclear factor-kappa B
LLO	Listeriolysin O
TEM	Transmission electron microscopy
NTA	Nanoparticle tracking analysis
BCA	Bicinchoninic acid
PBS	Phosphate-buffered saline
BHI	Brain heart infusion
DMEM	Dulbecco’s modified Eagle medium
FBS	Fetal bovine serum
CCK-8	Cell Counting Kit-8
qRT-PCR	Quantitative real-time polymerase chain reaction
IHC	Immunohistochemistry
HE	Hematoxylin and eosin
HRP	Horseradish peroxidase
PVDF	Polyvinylidene fluoride
TBST	Tris-buffered saline containing Tween-20
BSA	Bovine serum albumin
DAPI	4′,6-diamidino-2-phenylindole
iNOS	Inducible nitric oxide synthase
TNF-α	Tumor necrosis factor-alpha
MHC	Major histocompatibility complex
ANOVA	Analysis of variance
SEM	Standard error of the mean
