# Supplementary material for: Listeria monocytogenes-Derived Membrane Vesicles Suppress Melanoma Growth via Macrophage Activation Involving NF-κB Signaling
Source: Microorganisms. 2026 May 3;14(5):1038. doi: 10.3390/microorganisms14051038 (PMC13209653; doi:10.3390/microorganisms14051038)
Supplement: Supplementary file 1 [file microorganisms-14-01038-s001.zip › microorganisms-4271275_original_figure_images(1) (1).zip!/microorganisms-4271275_original_figure_images(1) (1)/original data/figure1/Fig1B/20250411_0014_100_size_488.pdf]

Electrophoresis & Brownian Motion
Video Analysis
Laser Scattering Microscopy

www.particle-metrix.de

Operator (Report): ZetaView

Video Operator: ZetaView

Sample Parameters

Sample Name: 100
Comment: Sample Remarks0:
Sample Remarks1:
Sample Remarks2:
Electrolyte:
Temperature: 28.36 °C sensed
pH 7.0 entered
Conductivity: 20.28 µS/cm sensed

Instrument Parameters

Laser Wavelength: 488 nm
Filter Wavelength: Scatter

Measurement Parameters

Cell S/N: ZNTA

Measurement Mode: Size Distribution 1 Cycle

11 Positions

Result (sizes in nm)

	Number	Concentration	Volume
Median (X50)	132.9	132.9	198.5
Span	54.0	54.0	110.3

Concentration: 6.8E+7 Particles / mL
Dilution Factor: 2000
Original Concentration: 1.4E+11 Particles / mL

Quality

Average Counted Particles per Frame: 195
Number of Traced Particles: 1074

Analysis Parameters

Max Area: 1000, Min Area: 10, Min Brightness: 30

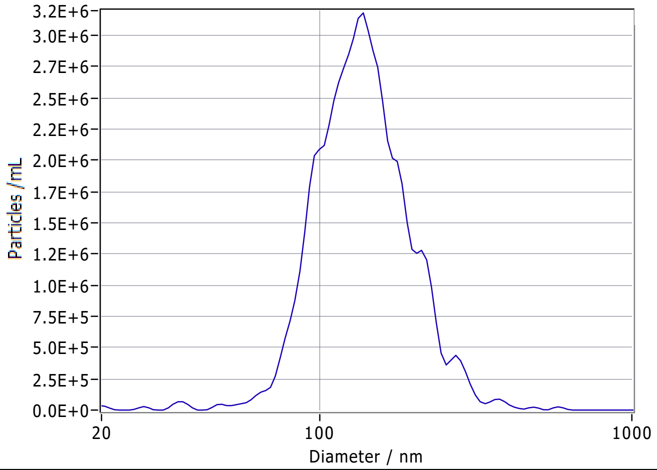

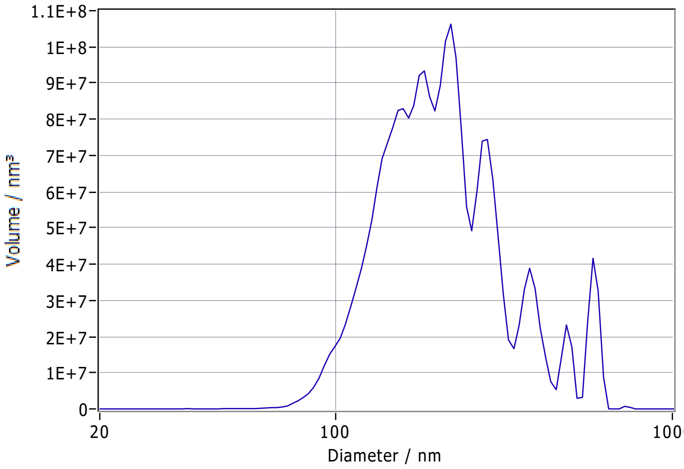

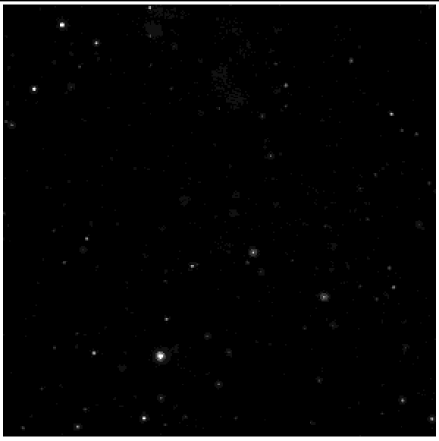

Peak Analysis (Concentration)

Diameter / nm	Particles/mL	FWHM / nm	Percentage
136.6	3.2E+6	97.6	100.0

X Values (all sizes are given in nm)

	Number	Concentration	Volume
X10	89.4	89.4	123.1
X50	132.9	132.9	198.5
X90	209.0	209.0	371.6
Span	0.9	0.9	1.3
Mean	144.9	144.9	229.7
StdDev	54.0	54.0	110.3

Comment

(Signature)

Analyzed Video: C:\320 ljh\4.11\20250411_0014_100_size_488.avi

ZetaVIEW S/N 20-524, Software ZetaView (version 8.05.12 SP1), Camera 0.713 mum/px

Experiment: 2025-04-11 17:28, Report: 2025-04-11 17:29
